# Nafamostat mesilate for anticoagulation in pediatric trauma patients undergoing extracorporeal membrane oxygenation: a two-center observational study in Northwest China

**DOI:** 10.3389/fmed.2026.1857646

**Published:** 2026-07-14

**Authors:** Weikai Wang, Zhe Lv, Haitong Wu, Hua Zhang, Zhen Chen, Yong Zhou, Zhangyan Guo, Jingmei Li, Le Ma, Dan Yao, Ying Wang, Ru Lin, Yajun Niu, Yuhao Ma, Xinxin Yan, Shan Jia, Taining Zhang, Yufei Wang, Yanqiang Du, Yi Wang

**Affiliations:** 1 Pediatric Intensive Care Unit, Gansu Provincial Maternity and Child-care Hospital, Gansu Provincial Central Hospital, Lanzhou, China; 2 Pediatric Intensive Care Unit, The Affiliated Children's Hospital of Xi'an Jiaotong University, Xi'an, China; 3 Department of Medicine, Xi'an Jiaotong University Health Science Center, Xi'an, China; 4 Graduate School, Xi'an Medical University, Xi'an, China; 5 Queen Mary School, Nanchang University, Nanchang, China

**Keywords:** anticoagulation, extracorporeal membrane oxygenation, heparin, nafamostat mesilate, pediatric

## Abstract

**Background:**

In recent years, accidental injury has emerged as a leading cause of mortality in the pediatric population. Such trauma frequently induces severe damage to parenchymal organs as a result of significant external mechanical force. In severe cases, death may occur rapidly when impaired cardiopulmonary function cannot be adequately sustained. Extracorporeal membrane oxygenation (ECMO) plays a critical role in supporting these patients. However, the selection of an anticoagulation strategy remains a decisive factor influencing clinical outcomes. This study aims to evaluate the comparative efficacy of regional anticoagulation using nafamostat mesilate vs. systemic anticoagulation with unfractionated heparin in pediatric patients with trauma undergoing extracorporeal membrane oxygenation (ECMO) support.

**Methods:**

In this study, 40 patients hospitalized due to severe trauma and received ECMO assistance were selected as the research subjects. According to the anticoagulation protocol, the patients were divided into the nafamostat mesilate group and the unfractionated heparin (UFH) group. Demographic data, hematological profiles, and coagulation parameters were systematically compared at baseline, 24, and 72 h after admission in patients receiving ECMO support. Clinical outcomes were evaluated based on the following criteria: transfusion requirements, use of coagulation-related agents, incidence of complications, and successful removal. The SPSS 21.0 statistical software was utilized for data statistics.

**Results:**

A total of 40 patients were enrolled in this study, 24 cases in the nafamostat mesilate group and 16 cases in the UFH group. There was no statistically significant difference in characteristics of blood cell classification and coagulation-related laboratory parameters at admission. Compared with the nafamostat mesilate group, the UFH group exhibited a higher rate of new-onset active bleeding, circuit thrombosis, and this difference was statistically significant (*P* < 0.05). In the UFH group, the incidence of mandatory anticoagulant discontinuation was significantly higher than that in the nafamostat mesilate group, attributable to a greater volume of active bleeding. Subgroup analysis patients who underwent surgery, new-onset active bleeding occurred in two of eight patients in the nafamostat mesilate group, with no interruption of anticoagulation, whereas severe new-onset active bleeding occurred in six of nine patients in the UFH group, requiring discontinuation of anticoagulation (all *P* < 0.05).

**Conclusion:**

In trauma patients during ECMO support, nafamostat mesilate achieves effective anticoagulation comparable to unfractionated heparin (UFH) while significantly lowering the risk of bleeding.

## Introduction

Unintentional harm has emerged as one of the primary reasons for the emergencies threatening children's lives in the context of economic development. The failure of vital organs is associated with severe injuries that lead to the failure of the cardiopulmonary system, reducing systemic circulation and oxygenation. This represents one of the direct causes of mortality (1). Extracorporeal membrane oxygenation (ECMO) is a treatment method in which cardiopulmonary function is substituted, and critically ill patients are provided with temporary circulatory and respiratory support to extend the time for resuscitation. Extracorporeal Life Support Organization (ELSO) has verified the clinical effectiveness of ECMO in the treatment of trauma and in the care of public health emergencies. Nevertheless, there are unique problems in anticoagulant strategy in trauma patients as some traditional anticoagulants may increase the chances of hemorrhages and create unfavorable clinical results (2, 3). Unfractionated heparin (UFH) as the most widely used conventional anticoagulant, is characterized by its low cost and clinical convenience in application with high inter-individual variation in response. However, while achieving a good anticoagulant effect, it poses a risk of depleting coagulation factors and platelets, which in turn heightens the risk of spontaneous bleeding. Patients with injury who are put on ECMO have a much higher risk of hemorrhage and it is more difficult to control. A potent and predictable anticoagulant, nafamostat mesilate works on different targets of serine proteases. Nafamostat mesilate is a synthetic serine protease inhibitor with a molecular weight of 539.58. Its pharmacological effects include: ① Anticoagulation: it inhibits the activity of thrombin and coagulation factors VIIa, Xa, and XIIa (4); ② Antifibrinolysis: it binds to plasmin and prolongs fibrinolysis time (5); ③ Antiplatelet activity: it inhibits platelet aggregation and promotes the disaggregation of aggregated platelets (6); ④ Antitrypsin activity: it inhibits free trypsin, trypsin bound to α2-macroglobulin, and phospholipase A2 (7); and ⑤ Other effects: it also inhibits the kallikrein-kinin system and the complement system (4). Given its rapid onset and significant anticoagulant effect, this drug holds an irreplaceable value in clinical scenarios where high-risk patients require potent anticoagulant intervention. Typical indications include patients who need extracorporeal membrane oxygenation (ECMO) support after trauma or major surgery, those with contraindications to heparin (such as heparin-induced thrombocytopenia) or who cannot tolerate it, and as a salvage treatment option when conventional anticoagulation fails and the risk of bleeding is controllable (8–10). This investigation conducted a retrospective analysis of the safety profile and therapeutic efficacy of nafamostat mesilate in pediatric trauma patients undergoing ECMO support.

## Methods

### Participants

**Figure 1:**
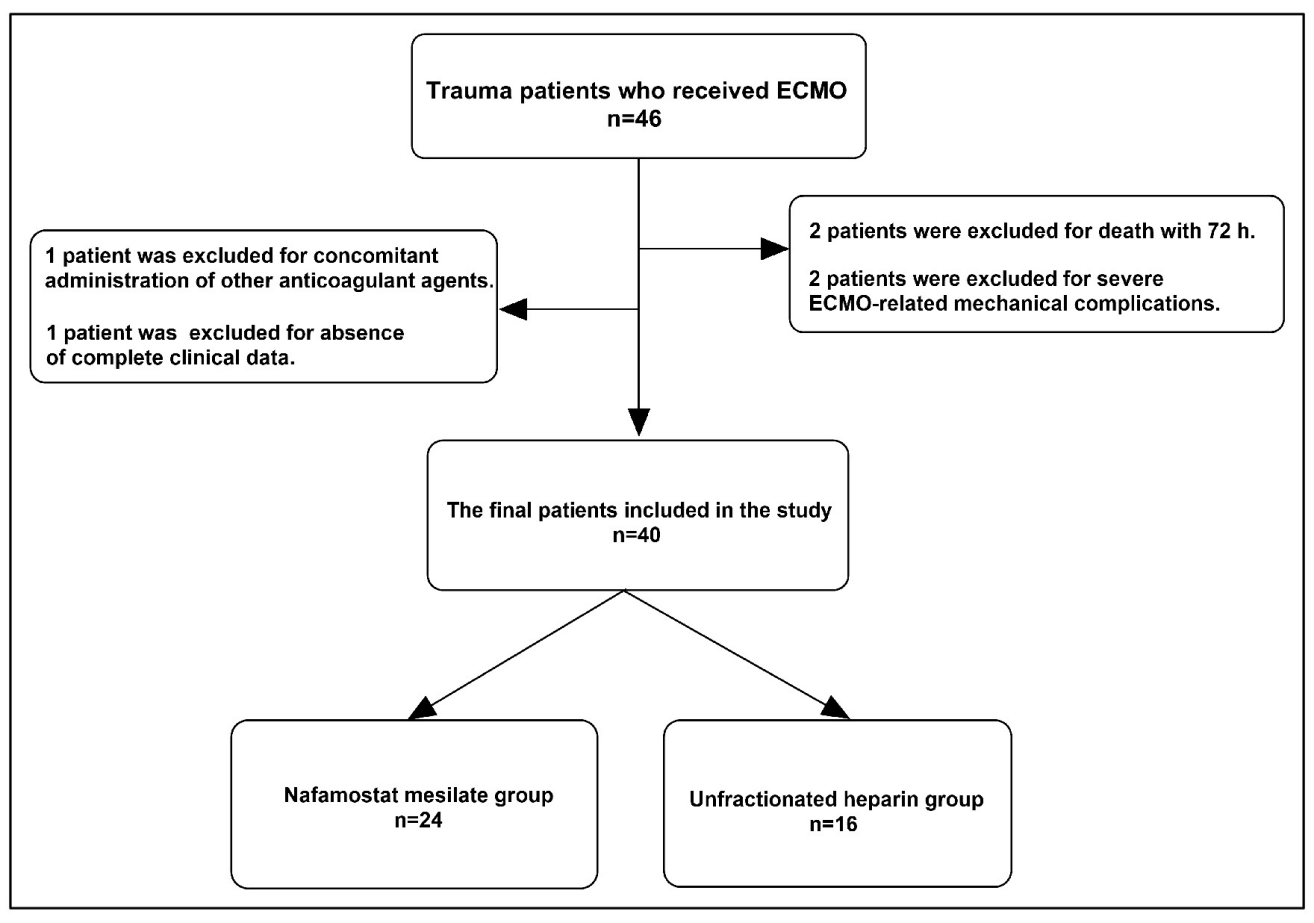
A flowchart of the study design.

In this study, 40 patients who received ECMO assistance and were hospitalized in the Pediatric Intensive Care Unit of Xi'an Children's Hospital and the Pediatric Intensive Care Unit of Gansu Provincial Maternal and Child Health Care Hospital from January 2021 to December 2025 due to severe trauma were selected as the research subjects. According to the anticoagulation protocol, the patients were divided into the nafamostat mesilate group (*n* = 24) and the unfractionated heparin (UFH) group (*n* = 16). Patients were excluded if they meet any of the following criteria: (1) death occurring within 72 h after initiation of ECMO support; (2) severe ECMO-related mechanical complications necessitating termination of ECMO therapy; (3) concomitant administration of other anticoagulant agents; or (4) absence of complete clinical data (Figure 1).

### Indications for ECMO

The patients who met any of the following criteria were considered to be eligible to receive veno-venous extracorporeal membrane oxygenation (VV-ECMO): (1) Severe respiratory failure: an arterial partial pressure of oxygen (PaO_2_) divided by fraction of inspiration of oxygen (FiO_2_) of less than 60–80 mmHg or an oxygenation index of more than 40; although the pediatric time limits have not been identified, practitioners can also use adult guidelines (PaO_2_/FiO_2_ < 60 mmHg over three or more hours, or PaO_2_/FiO_2_ < 80 mmHg over six or more hours). (2) Refractory response to standard treatments with mechanical ventilation and/or additional treatments such as high-frequency oscillatory ventilation, inhaled nitric oxide, or prone positioning. (3) Increased ventilator pressure parameters: mean airway pressure (MAP) >20–25 cm H_2_O with conventional ventilation, MAP >30 cm H_2_O with high frequency ventilation, or clinical diagnosis of ventilator induced lung injury. (4) Chronic respiratory failure with refractory respiratory acidosis (pH < 7.1) and/or hypoxia in spite of optimized ventilatory management (11).

The use of venous-arterial extracorporeal membrane oxygenation (VA-ECMO) was recommended in patients with the following clinical conditions: cardiac dysfunction not sufficient to support the appropriate circulation, which includes a consistent systolic blood pressure of less than 50 mm Hg, urine production of less than 1 ml/kg/h, confirmed lactic acidosis, systemic central venous oxygen saturation (ScvO_2_) of less than 0.60, or an arterial-to-venous oxygen saturation gradient greater than 0.30 in children with cyanotic congenital heart disease; accompanying neurological symptoms due to low cardiac output were also considered (12).

### Anticoagulation strategy

According to cardiopulmonary bypass anticoagulation protocols, 20 mg of nafamostat mesilate in 500 ml of 0.9% normal saline was used to prime the circuits in the nafamostat mesilate group. After extracorporeal circulation had been established, the drug was given at a rate of 0.2–0.5 mg/h/kg. Patients in the UFH group received an initial dose of heparin (20–50 U/kg) depending on the individual situation and then a continuous maintenance drip (5–10 U/kg/h). Both groups were kept at the targeted interval of 140–180 s of ACT in line with ELSO recommendations on anticoagulation management in pediatric extracorporeal life support (13).

### Data collection

Gather and evaluate these clinical parameters in each of the cohorts: age, sex, weight, classification of trauma, injury severity score, Glasgow Coma Scale (GCS), Lac-lactate, PaO_2_ /FiO_2_, Vasoactive-Inotropic Score (VIS), modes of medical intervention, mechanisms of injury, record of bleeding, and rates of surgery. Collect the actual values of blood cell analysis at admission including (leukocyte count, platelet count, hemoglobin, and hematocrit), as well as coagulation function-related indicators including (prothrombin time, activated partial thromboplastin time, thrombin time, fibrinogen concentration, and D-dimer) and the actual values 24 h and 72 h after ECMO assistance. The overall number of red blood cell suspension, plasma, platelets, fibrinogen, hemocoagulase, and prothrombin complex gathered per person in the course of the assistance was accumulated. Auxiliary time was recorded with a history of membrane lung failure, circuit thrombosis, embolic events and successful ECMO weaning.

### Assessment of hemorrhagic and thrombotic

The meaning of bleeding events is clearly defined as including the following: (1) bleeding caused by puncture or surgery, which requires surgical management; (2) intracranial hemorrhage; (3) gastrointestinal bleeding; and (4) bleeding in the respiratory tract that leads to the cessation or alteration of the original anticoagulation regime.

As per the given criteria, thromboembolic events are defined as follows: (1) The detection of an intracardiac thrombus with the help of an echocardiogram or the existence of thrombus formation in extracorporeal circulation circuitry; (2) Embolic stroke diagnosis based on computed tomography (CT) or magnetic resonance imaging (MRI); (3) Systemic thrombosis identified by diagnostic imaging methods regardless of the anatomical site or pathological type.

### Statistical analysis

The SPSS 21.0 statistical software was utilized for data statistics, and the Shapiro–Wilk test was applied to perform the normality test on continuous variables. Normally distributed continuous variables are presented as mean ± standard deviation (SD), and comparisons between groups were performed using the independent samples *t*-test. Categorical variables were represented as as number (percentage). Inter-group comparisons for categorical variables were performed using the Chi-square test or Fisher's exact test. Significant differences were indicated by *p* < 0.05.

## Results

### General characteristics of the two groups of patients

A total of 40 patients were enrolled in this study, including 24 in the nafamostat mesilate group and 16 in the UFH group. There were no statistically significant differences between the two groups in terms of gender, age, weight, primary extracorporeal circulatory support modality, injury severity score, GCS, Lac, PaO_2_/FiO_2_, VIS, types of traumatic injury, incidence of bleeding, Injuries site, rate of surgical intervention at admission, duration of mechanical ventilation, length of ICU stay, and length of hospital stay (Table 1). There was no statistically significant difference in Characteristics of blood cell classification and Coagulation-related laboratory parameters between the two groups of patients at the time of admission (Table 2).

**Table 1:** General characteristics of the two groups of patients.

	Nafamostat Mesilate group *n* = 24	Unfractionated heparin group *n* = 16	*p*-value
Gender			
Male (*n*, %)	14 (58.33)	9 (56.25)	0.512
Female (*n*, %)	10 (41.67)	7 (43.75)	
Age (y, mean ± SD)	9.6 ± 3.4	11.4 ± 5.6	0.262
Weight (kg, mean ± SD)	48 ± 25	54 ± 19	0.395
Injury severity score	19 ± 4	22 ± 5	0.055
GCS	7 ± 3	8 ± 3	0.309
Lac	4.5 ±2	5.1 ± 1.8	0.33
PaO_2_/FiO_2_	93 ± 21	102 ± 36	0.21
VIS	18 ± 3	20 ± 5	0.165
Extracorporeal circulatory support modality (*n*, %)			
VA-ECMO	8 (33.33)	5 (31.25)	0.891
VV-ECMO	16 (66.67)	11 (68.75)	
Types of trauma (*n*, %)			
Traffic accident injuries	18 (75)	11 (68.75)	0.664
Fall injuries	6 (25)	5 (31.25)	
Bleeding at admission (*n*, %)	12 (50)	9 (56.25)	0.698
Surgical intervention at admission (*n*, %)	8 (33.33)	9 (56.25)	0.151
Injuries sites (*n*, %)			
Severe thoracic trauma	8 (33.33)	5 (31.25)	0.891
Concomitant thoracic and abdominal trauma	10 (41.67)	9 (56.25)	0.179
Others	6 (25)	2 (12.5)	0.439^*^
Duration of mechanical ventilation (d, mean ± SD)	12 ± 5	14 ± 3	0.123
Length of ICU stay (d, mean ± SD)	21 ± 8	19 ± 10	0.509
Length of hospital stay (d, mean ± SD)	41 ± 18	39 ± 21	0.757

GCS, Glasgow Coma Scale; Lac-lactate; PaO_2_/FiO_2_-partial pressure of arterial oxygen/fraction of inspired oxygen; VIS, Vasoactive-Inotropic Score.

^*^Fisher's exact test.

**Table 2:** Laboratory test profiles of the two groups upon admission.

	Nafamostat mesilate group *n* = 24	Unfractionated heparin group *n* = 16	*p*-value
Characteristics of blood cell classification			
Leukocyte count (×10^9^/L, mean ± SD)	14 ± 7	13 ± 9	0.711
Platelet count (×10^9^/L, mean ± SD)	79 ± 21	64 ± 30	0.094
Hemoglobin concentration (g/L)	89 ± 31	76 ± 34	0.229
Hematocrit (%, mean ± SD)	24 ± 12	22 ± 10	0.571
Coagulation-related laboratory parameters			
Prothrombin time (s, mean ± SD)	19 ± 8	21 ± 9	0.478
Activated partial thromboplastin time (s, mean ± SD)	55 ± 21	63 ± 24	0.287
Thrombin time (s)	24 ± 11	23 ± 9	0.755
Fibrinogen concentration (g/L)	1.6 ± 0.6	1.4 ± 0.5	0.261
D-dimer (ug/ml)	6.5 ± 2.8	6.9 ± 3.1	0.681

### Relevant clinical indicators during the ECMO-assisted process

**Figure 2:**
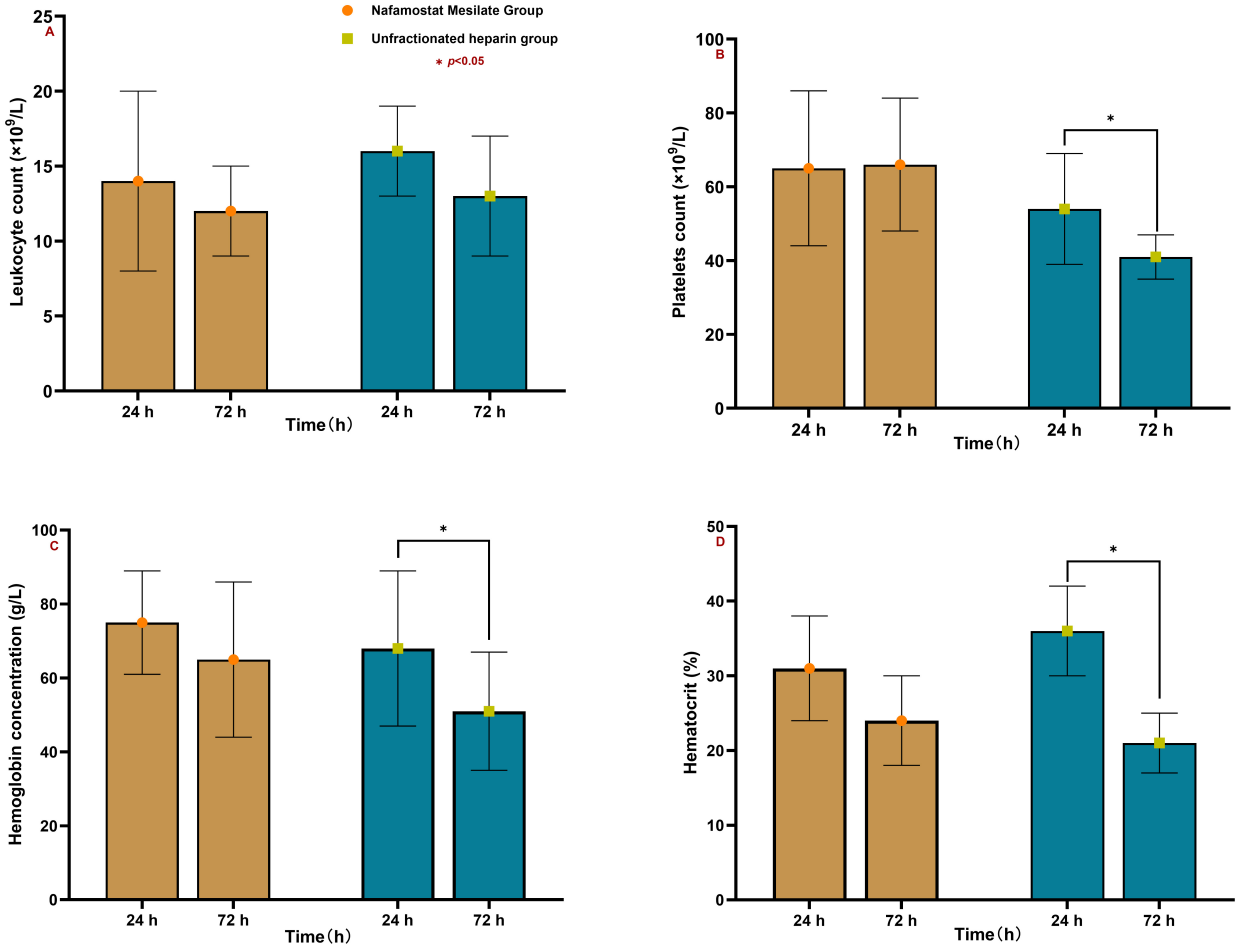
Temporal changes in blood cell classification in the two groups During ECMO. Comparison of the differences between the two groups of patients at 24 and 72 h in **(A)** Leukocyte count; **(B)** Platelets count; **(C)** Hemoglobin concentration; **(D)** Hematocrit.

**Figure 3:**
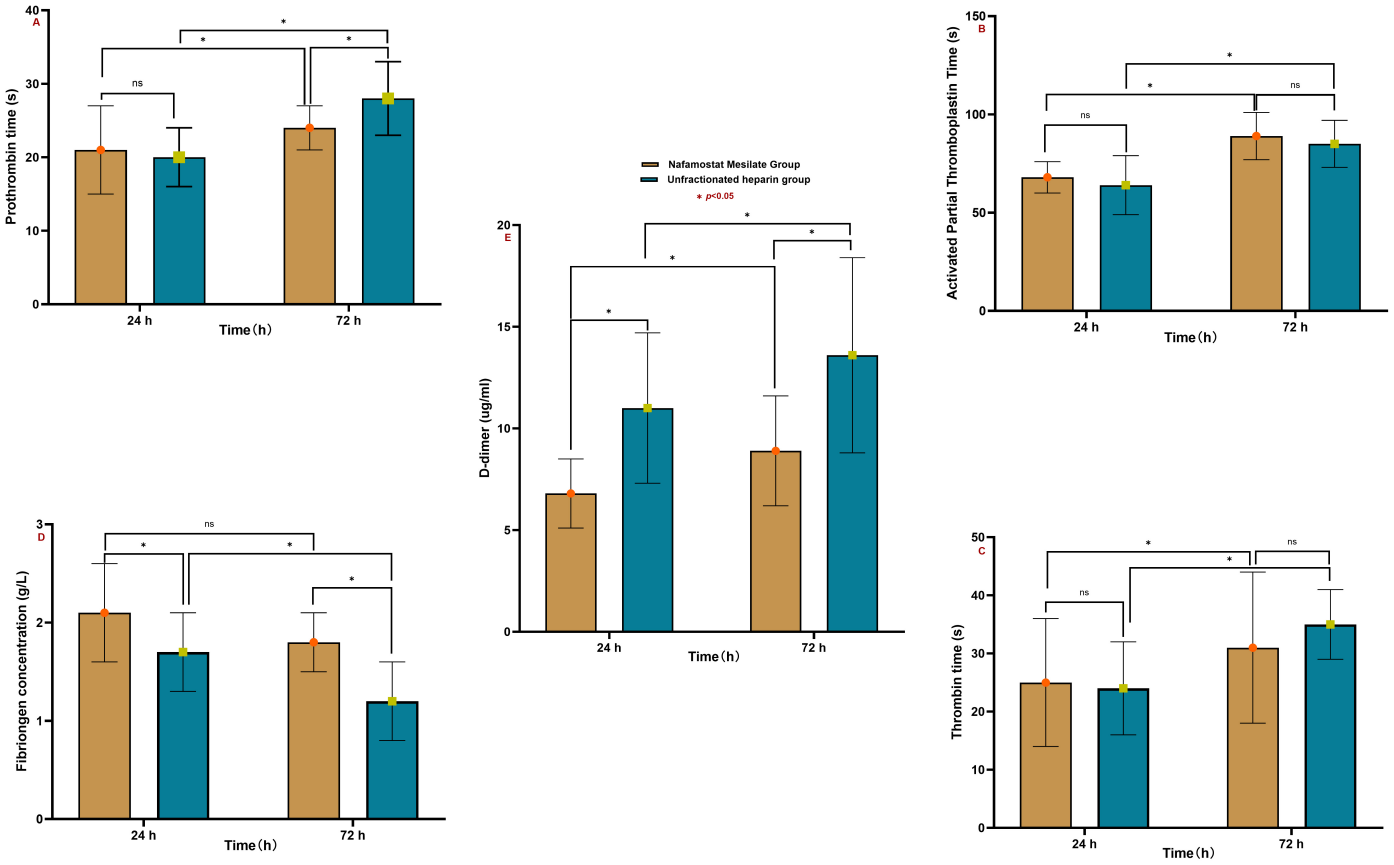
Temporal changes in coagulation-related laboratory parameters in the two groups during ECMO. Comparative analysis of intergroup and intragroup differences between the two patient groups at 24 and 72 h in anticoagulant efficacy in **(A)** Prothrombin time; **(B)** Activated partial thromboplastin time; **(C)** Thrombin time; **(D)** Fibrinogen concentration; **(E)** D-dimer.

There was no statistically significant difference in Leukocyte count at 24 and 72 h during the ECMO assistance process between the two groups of patients. Compared with the UFH group, Platelet count (×10^9^/L) at 72 h with (66 ± 18 vs. 41 ± 6, *p* < 0.001), hemoglobin concentration (g/L) at 72 h with (68 ± 21 vs.51 ± 16, *p* = 0.006), Hematocrit (%) at 24 h with (31 ± 7 vs. 24 ± 6, *p* = 0.005) and at 72 h with (36 ± 6 vs. 21 ± 4, *p* < 0.001; Figure 2). Compared with the UFH group, in the nafamostat mesilate group Prothrombin time (s) at 24 h (21 ± 6 vs. 27 ± 3) and at 72 h (20 ± 4 vs. 28 ± 5), and Activated Partial Thromboplastin Time (s) at 24 h with (68 ± 8 vs. 89 ± 12) and at 72 h with (64 ± 1 vs. 85 ± 12), Thrombin time (s) at 24 h with (25 ± 11 vs. 31 ± 13) and at 72 h with (24 ± 8 vs. 35 ± 6), D-dimer (ug/ml)at 24 h with (6.8 ± 1.7 vs. 8.9 ± 2.7) and at 72 h with (11 ± 3.7 vs.13.6 ± 4.8) were all decreased, the differences were statistically significant (all *p* < 0.05); and Fibrinogen concentration (g/L) at 24 h with (2.1 ± 0.4 vs. 1.8 ± 0.3, *p* = 0.023) and at 72 h with (1.7 ± 0.4 vs. 1.2 ± 0.4, *p* = 0.001) is increased, the differences were statistically significant (Figure 3).

### Cumulative blood product and coagulant transfusions per capita during ECMO support

**Figure 4:**
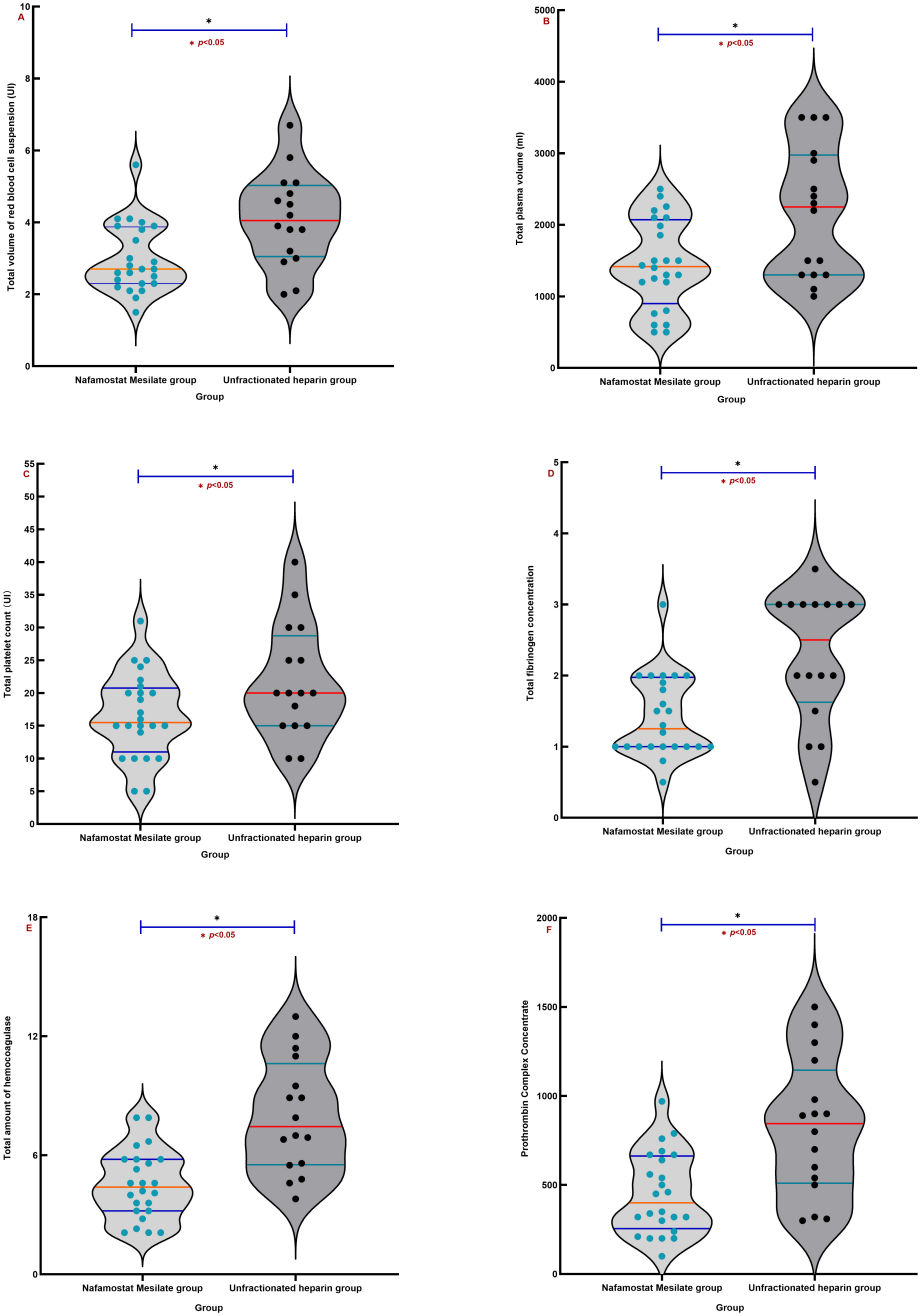
Characteristics of cumulative blood product and coagulant transfusions per capita during ECMO support. The cumulative quantities of blood product and coagulant transfusions in the two patient groups in **(A)** Red blood cell suspension; **(B)** Plasma; **(C)** Platelets; **(D)** Fibrinogen; **(E)** Hemocoagulase; **(F)** Prothrombin complex.

Compared with the nafamostat mesilate group, the UFH group had significantly higher mean cumulative transfusion volumes per patient during ECMO support, including red blood cell suspension (2.9 ± 0.89 vs. 4.23 ± 1.24, *p* = 0.001), plasma (1,476 ± 593 vs. 2,218 ± 794, *p* = 0.004), platelets (16.33 ± 6.27 vs. 22.19 ± 8.43, *p* = 0.025), fibrinogen (1.38 ± 0.58 vs. 2.25 ± 0.85, *p* = 0.002), hemocoagulase (4.56 ± 1.61 vs. 8.16 ± 2.85, *p* < 0.001), and prothrombin complex (460 ± 216 vs. 815 ± 416, *p* = 0.005; Figure 4).

### Outcomes

**Figure 5:**
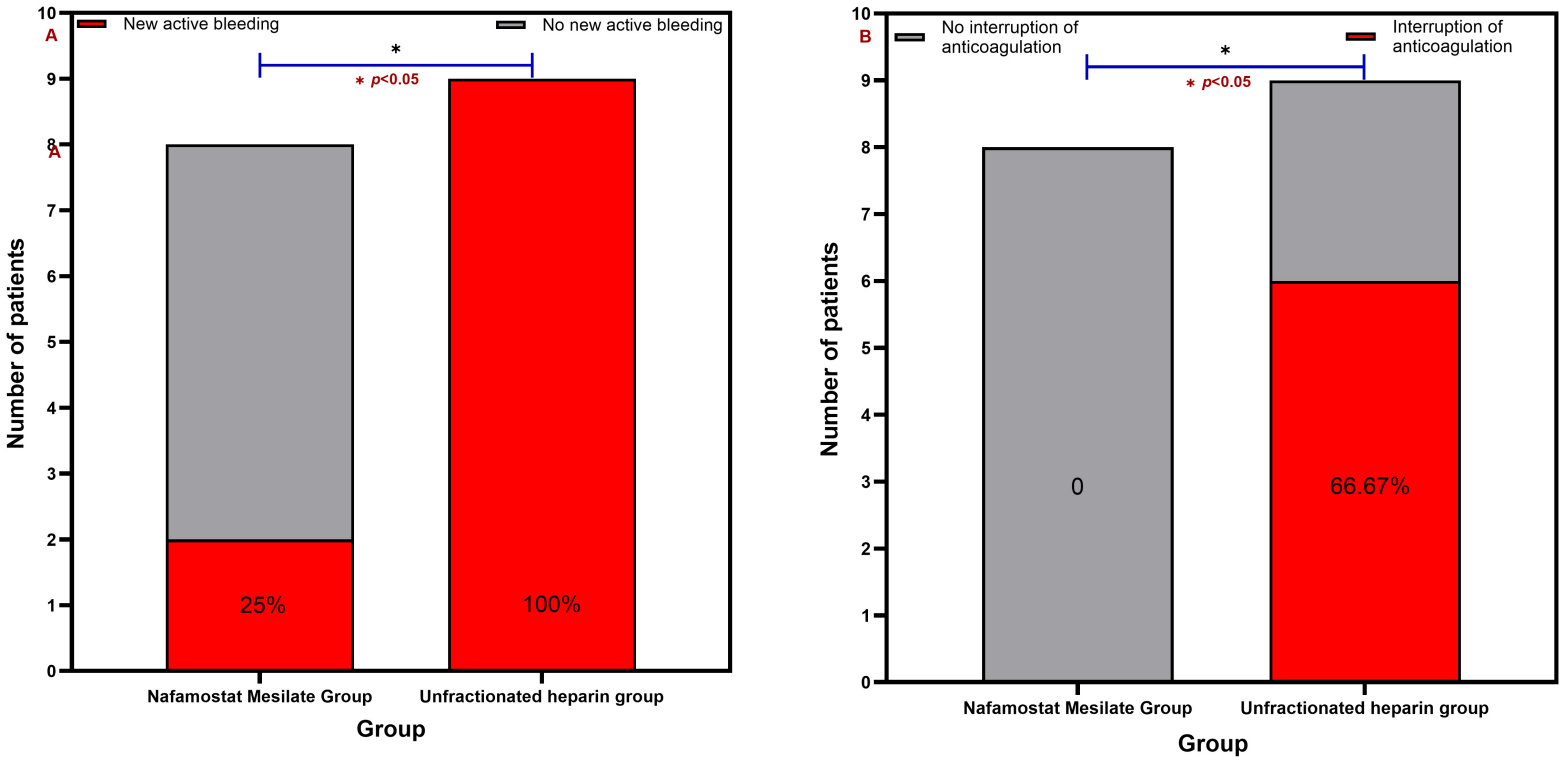
Characteristics of rebleeding during ECMO support in the subgroup that received surgical intervention at the time of admission. **(A)** illustrates the incidence of recurrent bleeding in the two patient groups within the surgical intervention subgroup, **(B)** depicts the proportion of patients with recurrent bleeding who required interruption of anticoagulation therapy. *Fisher's exact test.

Compared with the nafamostat mesilate group, the UFH group had significantly higher incidences of new-onset active bleeding and circuit thrombosis (*p* < 0.05). The rate of mandatory anticoagulation interruption due to massive active bleeding was also significantly higher in the UFH group than in the nafamostat mesilate group (Table 3). There were no significant between-group differences in ECMO support duration, oxygenator dysfunction, embolism, or successful weaning rate (Table 3). In the subgroup that received surgical intervention at the time of admission recurrent bleeding occurred in two of eight patients in the nafamostat mesilate group without anticoagulation interruption, while severe recurrent bleeding occurred in six of nine patients in the UFH group requiring anticoagulation discontinuation, and all differences were statistically significant (all *p* < 0.05; Figure 5).

**Table 3:** Clinical outcomes and complications in the two groups.

Clinical outcomes and complications	Nafamostat mesilate group *n* = 24	Unfractionated heparin group *n* = 16	*p*-value
New-onset active bleeding (*n*, %)	2 (8.33)	9 (56.25)	0.003^*^
ECMO-assisted time (*d*, mean ± SD)	6.8 ± 3.5	11.5 ± 6.7	0.055
Membrane lung dysfunction (*n*, %)	1 (4.17)	3 (18.75)	0.289^*^
Circuit thrombosis (*n*, %)	2 (8.33)	9 (56.25)	0.003^*^
Interruption of anticoagulation (*n*, %)	0	6 (37.5)	0.003^*^
Successful removal (*n*, %)	23 (95.83)	14 (87.5)	0.553

^*^Fisher's exact test.

## Discussion

ECMO is being increasingly employed in the clinical emergency treatment of pediatric trauma, highlighting the pressing necessity for standardized, evidence-based clinical practice guidelines that are founded on high quality research and intended for practical implementation. Patients experiencing acute cardiopulmonary failure secondary to trauma are associated with an increased risk of adverse neurological outcomes including severe brain dysfunction and higher mortality rates (14, 15). Severe thoracic trauma often manifests in acute hemorrhage and impaired pulmonary performance. The clinical results are still not satisfactory despite the aggressive therapies such as emergency thoracotomy and resuscitation, and they are often associated with significant surgical trauma and minimal clinical advantages. Approximately only 30% of these patients will regain spontaneous circulation, the total survival rate is as low as 6% (1.6% in case of blunt trauma and 10.2% in case of penetrating trauma) (16).

Thoracic trauma is a significant cause of serious injury in children. Moreover, the cardiopulmonary function may become worse in these patients after the injury. The timely administration of ECMO offers an important bridge to recover the native cardiopulmonary function or to provide a definite surgical intervention and thereby avoids the complications associated with prolonged hypoxia. Since conventional resuscitation approaches have proven to be relatively ineffective in critically injured children, there has been growing literature and clinical reports identifying ECMO as a rescue therapy in pediatric trauma (16–19).

ECMO can be considered a critical life-support mechanism used in treating severely ill children. However, its use in the clinic presents significant difficulties because of specific physiological characteristics of the children, particularly their natural coagulation profiles. The two most typical complications are bleeding and thrombosis that directly affect clinical outcomes in pediatric ECMO (20, 21). It is especially true in case of severe trauma, when patients can have solid organ rupture, traumatic subcutaneous bleeding, or even hemorrhagic shock caused by continuous bleeding. Moreover, the majority of patients are found to have not only cardiopulmonary failure but also simultaneous hemorrhage and damage to other parts of the body which often necessitate emergency intervention. Anticoagulation is necessary in the course of ECMO support, however, there is one additional layer of complexity involved in management in this high-risk group of patients.

UFH has a rapid onset of action (around 3–5 min), but a long half-life (about 0.5–3 h in adults and possibly more than that in children because of their developmental pharmacokinetics) (22, 23). Although systemic anticoagulation is necessary to avoid circuit thrombosis, it simultaneously raises the likelihood of bleeding, which is especially problematic with injured children, who are particularly vulnerable to severe hemorrhage. The use of heparin can also result in coagulopathy by using up clotting factors and reducing the number of platelets. Hence, anticoagulation management is one of the critical clinical problems of pediatric trauma patients on ECMO. Even though new anticoagulants and heparin-free methods are under research, balancing the risks of thrombosis and bleeding has remained challenging.

In the current research, severe coagulation disorder was recorded in the UFH group 24 h following the start of ECMO. Particularly, PT and APTT, thrombin time (TT) and D-dimer levels had a significant increase, whereas the fibrinogen concentration, number of platelets, and hematocrit decreased which contributed to an increase in bleeding risk. The incidence of bleeding was therefore significantly higher in the UFH group than in the nafamostat mesilate group, requiring additional transfusions of blood product and coagulation factor. In spite of no statistically significant mortality difference between the two groups, ECMO management was significantly more complicated in the UFH group, which added to the clinical burden (23).

Nafamostat mesilate is a non-natural serine protease inhibitor capable of selectively inhibiting diverse coagulation-related enzymes such as thrombin, activated coagulation factors XIIa and Xa, complement fractions C1r and C1s, plasmin, trypsin, and kallikrein, and thus efficiently inhibits the activation of the coagulation system (19–26). Because it had a short half-life, this drug was first introduced and licensed purely as an anticoagulating medicine in continuous hemofiltration (27, 28). As an alternative anticoagulant to heparin in patients undergoing extracorporeal membrane oxygenation (ECMO), nafamostat mesilate has been used in various studies to reduce bleeding complications, and has shown good results in preventing bleeding (28). Nafamostat mesilate had particular benefits in patients who were actively bleeding or at risk of bleeding. In the present research, the bleeding rate was significantly decreased in the nafamostat mesilate group over the heparin group. Also, the demand of blood components and coagulation factors was less, which indicates very good clinical safety and efficacy and is consistent with previous reports (29–32).

Although the advantage of nafamostat mesilate in reducing bleeding risk during ECMO support is widely acknowledged, there remains a lack of objective and scientific validation through randomized controlled trials comparing it with UFH. This is particularly true for pediatric populations, where fewer relevant studies exist. While this study is limited by its retrospective design and small sample size, it nonetheless provides an objective assessment of the benefits of nafamostat mesilate in trauma patients undergoing ECMO support, offering valuable insights for clinicians. Additionally, because multiple comparisons were performed without correction for multiplicity, the findings should be considered exploratory. Future prospective, multicenter, randomized controlled trials with larger sample sizes and pre-defined multiplicity adjustments are warranted to confirm these results.

## Conclusion

For trauma patients on ECMO, anticoagulation with nafamostat mesilate is linked to a lower incidence of hemorrhagic complications and a reduction in blood product transfusion volumes.

## Data Availability

The raw data supporting the conclusions of this article will be made available by the authors, without undue reservation.
